# Reconstructing charcoal formation temperatures in archaeology and volcanology using an automated 532 nm Raman spectroscopy approach

**DOI:** 10.1038/s41598-026-53711-0

**Published:** 2026-05-23

**Authors:** Fabian Dellefant, Olivier Brückner, Julia Budka, Claudia A. Trepmann, Fabio Joseph, Giulia D’Ercole, Kaja M. Schultz, Anna Huber, Werner Ertel-Ingrisch, Bettina Scheu, Paul A. Wallace, Karen Fontijn, Melanie Kaliwoda

**Affiliations:** 1 Department of Cultural and Ancient Studies, Ludwig-Maximilians-Universität München, Katharina-von-Bora-Str. 10, 80333 Munich, Germany; 2 Department of Earth and Environmental Sciences, Ludwig-Maximilians-Universität München, Theresienstr. 41, 80333 Munich, Germany; 3 Interaction Design, Hochschule für Gestaltung Schwäbisch Gmünd, Rektor-Klaus-Str. 100, 73525 Schwäbisch Gmünd, Germany; 4 Mineralogical State Collection Munich, Bavarian Natural History Collections, Theresienstr. 41, 80333 Munich, Germany; 5 Department of Geosciences, Environment and Society, Université libre de Bruxelles, Avenue Franklin Roosevelt 50, 1050 Brussels, Belgium

**Keywords:** Raman spectroscopy, Charcoal temperature calibration, Open-access tool, Statistical analysis, Ceramics, PDC temperature, Chemistry, Materials science

## Abstract

Raman spectra of charcoal provide structural information that enables the reconstruction of past combustion conditions. We present a calibration method (532 nm laser excitation) for determining charring temperatures from Raman spectra of amorphous carbon, based on Raman band intensity ratios (HD/HG). Using a pine wood reference dataset, we establish statistical criteria for estimating temperature, identifying sp²-hybridization clustering, and detecting oxidative weathering. To ensure reproducibility and accessibility, we introduce our tool CHARM as a free, automated webpage (https://olivierbrcknr.github.io/charm/) for processing Raman data—including de-noising, baseline correction, parameter extraction, and temperature reconstruction. This tool generates standardized numerical and graphical outputs that drastically reduce processing time and analytical bias. Applications to archaeological ceramics demonstrate that reliable temperature estimates of blackened surfaces can be achieved without destructive sampling, while tests on thin section preparations confirm that Raman parameters remain unaffected. Furthermore, our protocol enables statistical analysis of charcoals from volcanological contexts, revealing interpretable temperature ranges despite charcoal modifications by oxidative weathering. Our calibration provides a robust method for consistent, rapid temperature reconstruction of amorphous carbon across archaeological, volcanological, and related fields.

**Supplementary Information:**

The online version contains supplementary material available at 10.1038/s41598-026-53711-0.

## Introduction

Charcoal, the carbon-rich residue formed by the incomplete combustion of organic material, is a critical material in both archaeology and geology due to its unique capacity to preserve information^1^. In archaeological contexts, charcoal serves as a direct record of past human activities, such as ceramics production^2,3^, fuel use^4^, and land management^5,6^. Its anatomical structure allows for the identification of plant taxa, supporting reconstructions of ancient environments, and resource exploitation^7^. Moreover, charcoal is a key material for radiocarbon dating^8^, providing chronological frameworks for archaeological sites and cultural sequences^9^. In geology, and particularly in volcanology, charcoal embedded within volcanic ash deposits offers valuable evidence for the timing^10^ and environmental impact of eruptive events^11^. Charcoal’s resistance to decay under many burial conditions makes it a robust archive for both archaeology and volcanology^12,13^.

Beyond its role as a chronological and paleoenvironmental marker, charcoal also records the temperature conditions under which it was formed^14^. The thermal history of charcoal—specifically, the maximum temperature and duration of exposure during its formation—affects its molecular structure and chemical composition^15–18^. Raman spectroscopy has emerged as a powerful, non-destructive tool for probing the microstructural and chemical changes in charcoal related to its formation temperature^2,19^. This technique analyzes the vibrational modes of carbon atoms in the charcoal matrix, which correlate with the degree of thermal alteration^15,16^. Charcoal Raman spectra typically exhibit two bands: the defect band (D-band) at ≈ 1360 cm^− 1^ and the graphite band (G-band) at ≈ 1600 cm^− 1^, along with a valley region (V-valley) at ≈ 1450 cm^− 1^^2^. The D-band is associated with the vibrational modes of disordered or edge regions in sp²-hybridized carbon systems, particularly at the edges of aromatic rings, whereas the G-band arises from the in-plane bond-stretching vibrations of all sp²-hybridized carbon atoms in both rings and chains^20^. During the pyrolysis or carbonization of wood, the intensity or height (H) of the D-band in the Raman spectrum increases monotonically with increasing carbonization temperature, typically within the range of 500–1200 °C^15,20,21^. Higher temperatures promote the formation and growth of aromatic clusters (sixfold rings), which enhance the D-band signal^20^. The intensity ratio of the D-band and the G-band (HD/HG) also depends on the excitation laser wavelength used during Raman measurements. Calibrations have been established for excitation wavelengths of 514.5 nm^15^, 635 nm^17^, and 785 nm^16^, enabling temperature reconstruction from Raman data under controlled laboratory conditions. However, these calibrations cannot be directly applied across different excitation wavelengths, because the D- and G-bands exhibit wavelength-dependent intensity and position variations that alter the measured HD/HG ratio^22^. The 532 nm excitation results in an optimal signal-to-noise ratio for amorphous carbon^2^, yet no systematic HD/HG–temperature calibration currently exists for 532 nm data, preventing direct comparison of results obtained under this configuration with previously published calibrations. Therefore, a dedicated 532 nm calibration is required to enable reproducible and accurate interpretation of charcoal Raman spectra across laboratories using this wavelength.

For this study, we build directly on the methodological framework proposed for the 514.5 nm calibration^15^ by Damien Deldicque, Jean Noël Rouzaud, and Bruce Velde for carbonization temperatures and incorporate the recent findings about the influence of oxidative weathering^23^ on charcoal Raman parameters. We present an HD/HG ratio–temperature calibration for Raman spectra acquired with a 532 nm excitation laser, developed to quantify Raman parameters in unweathered charcoal. We test this calibration on different wood types charred under bonfire conditions, recently produced charcoal in ceramics, and charcoal formed and buried by pyroclastic density current (PDC) following a volcanic eruption. In general, data processing steps, including baseline correction and smoothing, are time-consuming and can considerably influence the parameters extracted from Raman spectra of carbonaceous materials^15,24,25^. Consequently, direct comparisons of Raman parameters, such as quantifying absolute firing temperatures of carbon-bearing samples, require that both calibration and sample Raman spectra undergo identical processing and data extraction procedures^22^. Failure to do so poses a significant risk of temperature misinterpretation. Our methodological approach refines the existing calibration processes^15–17^ by incorporating an automated and standardized data treatment protocol, ensuring that both reference and sample data undergo identical baseline correction, smoothing, and parameter extraction procedures. This consistency is critical for avoiding processing-related discrepancies that can obscure temperature reconstructions.

Our tool, CHARM (Characterizing Amorphous carbon with Raman Measurements), is freely accessible via a dedicated webpage (see Abstract and Methods). Users can upload Raman data, which are automatically de-noised, baseline-corrected, and statistically compared to our calibration dataset. Results can be downloaded in tabular format, as well as in image and vector graphic forms. By providing an open-access, wavelength-specific (532 nm) calibration and automated processing workflow, this study promotes standardization of Raman-based temperature determinations for amorphous carbon and enables reliable, reproducible applications across archaeological, geological, and experimental research contexts.

### Carbonization

This process refers to the thermal decomposition of organic materials in an inert atmosphere, resulting in the loss of heteroatoms, including oxygen and hydrogen, and the formation carbon-rich solid residues. Carbonization is primarily governed by the maximum temperature applied in the range from 300 to 400 °C up to about 1200° C^26^. The type of carbon formed during carbonization depends on the nature of the organic precursor: oxygen-rich precursors, such as carbohydrates (including cellulose-based materials) typically yield non-graphitizing carbons, whereas hydrocarbon precursors tend to form graphitizing carbons^27,28^. Importantly, graphitization is distinct from carbonization and generally occurs at temperatures above 1200 °C. Heating organic precursor induces nanoscale restructuring, generating various reaction products. At temperatures below 600 °C, carbonization predominantly produces char and oil; at higher temperatures, char and gas become the dominant products^17^. Increasing both the temperature and the residence time leads to a more ordered char structure, approaching a graphite-like arrangement^16,29^. These structural changes can be analyzed using (micro-)Raman spectroscopy, which is particularly sensitive to sp² carbon structures^30^. The first-order sp² carbon-related D- and G-bands are well-documented in literature^22,24,25,31–33^.

### Previous calibration studies on charcoal formation temperatures using Raman spectroscopy

Various approaches using Raman spectroscopy have been employed to determine the maximum firing temperature of charcoal. For example, oxygen-rich and thus non-graphitizable carbons (wood, leaf fibers, sucrose crystals, and graphene oxide) were subjected to heat treatments up to 1000 °C and subsequently measured using a 785 nm excitation laser^16^. Following smoothing and photoluminescence slope subtraction^34^, a systematic structural evolution was observed. The results indicate that the intensity ratio of the V-valley and the G-band (HV/HG) decreases between 400 °C and 1000 °C^16^. The structural changes in organic materials can also be quantified using the HD/HG ratio, which increases with temperature as a result of structural changes and the size of sp²-hybridized carbon clusters^15–17,30^. A systematic investigation of the structural evolution during pyrolysis of various organic precursors, such as saccharose and wood, was conducted using a 514.5 nm excitation laser in combination with high-resolution transmission electron microscopy (HRTEM)^15^. Firing residence time (1 h, 6 h, and 12 h) is reported to have a minimal impact on structural changes, and differences due to precursor material were negligible^15^. As a result, a clear temperature dependence of HD/HG between 600 °C and 1300 °C was reported. The pyrolysis of beech wood was examined, employing Raman spectroscopy with a 635 nm laser alongside complementary techniques, including scanning electron microscopy (SEM) and Fourier-transform infrared spectroscopy (FTIR), to study structural, morphological, and surface chemistry changes^17^. With increasing temperatures, the ratio of the V-valley and the D-band height (HV/HD) and HV/HG decrease, while HD/HG increases.

However, a comparison of ancient chars to experimentally oxidized recent chars documented that weathering-induced oxidation of chars can distort spectra and might lead to an overestimation of maximum firing temperatures^23^. The position of the D-band combined with HD/HG was proposed to be able to distinguish between unaltered and altered char domains. Moreover, unaltered material correlates with low calcium concentrations at the micrometer-scale, based on energy-dispersive spectroscopy mapping from SEM data^23^. A 532 nm excitation laser was applied in a study to differentiate Late Bronze Age Nubian- and Egyptian-style ceramics (c. 1550–1400 BCE) from northern Sudan. The results indicate that Egyptian-style ceramics were fired at higher temperatures than Nubian-style ceramics. Moreover, it was reported that weathering systematically obscured the Raman data, as observed in the comparison of samples from the two localities, Sai Island and Dukki Gel^2^.

Several research groups have pointed out that relying on the positions of the D- and G-bands, or on HD/HG ratios, may not be the most reliable approach for evaluating the degree of disorder/order of carbon materials. Instead, these researchers argue for parameters derived from the width of the G-band as more effective indicators of the decrease in structural disorder^32,35^. However, in our study, G-band width measurements were not performed because the G-band often exhibited significant overlap with the D-band, as similarly discussed^15,16^. Therefore, to maintain methodological simplicity and consistency, deconvolution and curve-fitting procedures were intentionally avoided.

## Methods and sampling

### Raman spectroscopy

Micro-Raman spectroscopy was conducted at the Munich Mineralogical State Collection (MSM; within the *Staatliche Naturwissenschaftliche Sammlungen Bayerns*,* SNSB*) using a HORIBA JOBIN YVON XploRA ONE system equipped with a UI-3580LE-C-NO IDS camera. The Raman spectrometer has a Peltier-cooled CCD detector, a 2ω-Nd: YAG laser, and edge filters. A 532 nm wavelength laser was operated in an attenuated mode at 1% laser power combined with an 1800 g/mm grating, resulting in a power of approximately 57 µW on the sample surface without oxidizing and destroying the amorphous carbon. The hole and slit diameters were set to 300 μm and 100 μm, respectively, with an integration time of 2 × 10 s applied. On the sample surface, a 100× long working distance objective resulted in a 0.9 μm laser spot size. The spectra were measured using the HORIBA LabSpec 6 Spectroscopy Suite Software 6.7.2.11 (https://www.horiba.com/int/scientific/products/detail/action/show/Product/labspec-6-spectroscopy-suite-software-1843/). Wavelength calibration was performed using a pure Si-wafer chip on the predominant 520 ± 1 cm⁻¹ peak. The precision of the Raman peak shift position is estimated to be ± 1.5 cm⁻¹.

### Charcoal synthesis for calibration

A series of pinewood-based (*Pinus sylvestris*) chars were produced at 414, 511, 597, 655, 706, 755, and 803 °C in an autoclave setting and at 800, 900, 1000, 1100, and 1200 °C in a 1-atm gas mixing oven. Two different setups were chosen to ensure precise temperature control. As starting material, wood cubes (10–20 mm wide and high) were heated at the desired temperature for ≈1 h.

### 1-atm gas mixing oven setup

Experiments were performed in a GERO (Neuhausen) HTRV 70–2501 1-atm vertical gas mixing oven, equipped with a vertical muffle tube made of AL23 FRIATEC-Friedrichsfeld, which was encapsulated gas-tight against the lab atmosphere by two cooling heads and rubber seals within. For oxygen fugacity (*f*O_2_) control, a system of 6 WAGNER^®^ gas mixing valves was used to guarantee stable mass flows (max. 400 sccm/min) of pure Ar (99.999%). Before the performed experiments, the temperature profile of the position of the Pt crucible within the muffle tube was determined using a Pt–Rh type 1 thermocouple, resulting in a temperature uncertainty of ± 5 °C over 10 cm absolute length coverage of the entire setup^36^. Experiments were initiated after pre-setting the desired experimental conditions, including temperature and *f*O_2_, and allowing the furnace to equilibrate for ≈ 1 h. The wood cubes were positioned inside a Pt synthesis crucible (18 mm lower width; 30 mm upper width; 36 mm height), equipped with an Al_2_O_3_ support block machined according to the inner shape of the Pt synthesis crucible. After completion, the run products were retrieved from the furnace by lifting the Pt synthesis crucible from the furnace muffle tube using a steel hook and transferring the complete setup instantaneously into a desiccator for cooling to ambient temperatures.

### Autoclave setup

The carbonization experiments were conducted in a split-tube (Carbolite EST 12/60/300) modified fragmentation setup^37^ benefiting from a precise temperature control (ΔT = ± 5 °C) over 10 cm absolute height, covering the entire setup in its center, and an Ar-atmosphere (99.999%). The wood cubes were placed in a steel crucible and mounted in the center of a 40 cm long autoclave. The autoclave was tightly sealed at both ends; at the top, two small tubes are implemented; one is connected to an Ar line, and the second is used as exhaust. After closure, the autoclave was flushed at least 3 times with Ar under a mild overpressure to replace the air in the autoclave. A split-tube furnace was placed around the autoclave, heating the sample to the desired temperatures. A K-type thermocouple directly below the sample inside the autoclave enabled us to precisely monitor the sample temperature. Once the experimental temperature was reached, the furnace was turned down to stabilize the temperature. To end the experiment, the furnace was removed, and the autoclave was quenched with compressed air. After cooling, the carbonized sample was recovered.

### Anthropogenic and volcanological samples

To initially test our calibration and subsequently apply it in volcanological and archaeological contexts, additional charcoal samples were produced and selected. To broaden the calibration to different wood types, bonfires were constructed using spruce (*Picea abies*) and beech (*Fagus sylvatica*) wood. These fires were thermally monitored with a Sharplace 100 mm Spade K-type thermocouple (ΔT = ± 10 °C). For the analysis of charcoal within silicate environments, a ceramic sample was produced in 2025 using clay from the Ziegelwerk Lizzi (N47.71965814769331, E16.223041911537322) in Bad Erlach, Austria. The same pine wood used in the calibration served as the temper material. Sample Abri was produced in 2023 from Nile clay sediments and organic components collected in the village of Abri, Sudan, and fired in a bonfire setting. The sample was subsequently processed into a thin section (≈ 25 μm thickness) and examined using a Leica DM2700 P polarizing microscope under both reflected and transmitted light. Images were captured using a Leica MC170 HD camera and processed with Leica Application Suite X software 3.08.19082 (https://www.leica-microsystems.com/c/gl/las-x-software-registration/). Raman measurements were carried out on the thin section surface before and after an additional chemo-mechanical polish with a colloidal silica solution (Syton-method)^38^, to investigate the influence of thin section preparation and polishing on charcoal Raman spectra. A charcoal sample (OLK21-035D1) was collected from the Olkaria Volcanic Complex (Kenya) in 2021, extracted from the base of a PDC deposit approximately 2 km east of the most recently active Ololbutot vent in the Ol Njorowa Gorge^10^(Fig. 5a, b). Using the charcoal, a calibrated radiocarbon age of 191 ± 23 cal yr BP (i.e., 1759 ± 23 CE) was obtained^10^, constraining the timing of the most recent eruptive activity at Olkaria—the most geothermally productive site in Africa.

### Data processing

The following description of data processing was first coded as python scripts. After completion of the calibration, we translated the scripts to JavaScript and made them accessible on a webpage (https://olivierbrcknr.github.io/charm/), where Raman data can be uploaded and processed automatically as detailed.

For de-noising, we used the Savitzky–Golay (SG) filter^39–41^ and an already available script (https://www.npmjs.com/package/ml-savitzky-golay-generalized). Its primary purpose is to de-noise data while preserving essential features such as peak heights and widths. The method achieves this by fitting a low-degree polynomial function to a sliding window of data points using linear least squares, then replacing the central point with the polynomial function’s value, which is repeated across the whole dataset^39^. Additionally, the SG filter can compute derivatives of the data by differentiating the fitted polynomial function, enabling simultaneous smoothing and differentiation in a single computational step^40^. However, the method has limitations, particularly at data boundaries where asymmetric windows or extrapolation techniques are required to mitigate edge artefacts. By using the modified SG approach^42^, we bypass border problems and do not invent data points in the spectra.

We use the Eilers and Boelens baseline correction method^43^ with modifications^44^, which employs an asymmetric least-squares (ALS with parameters: *λ* = 1e5 and *p* = 1.5e-3) framework augmented with derivative-based peak screening to address the challenge of separating broad, overlapping Raman peaks from smooth luminescence baselines. This technique is particularly effective for spectra with extended peak tails, such as those observed in disordered carbon-based materials, including amorphous carbon. The algorithm smooths the raw spectrum using 16 successive iterations of mollification, implemented as a convolution operation. This step suppresses high-frequency noise while preserving peak morphology.

For automated band and valley detection, we define the D- and G-bands as maxima between 1300 and 1450 cm^-1^ and 1500–1650 cm^-1^, respectively, whereas the V-valley is defined as a minimum between 1400 and 1550 cm^-1^. Following data processing, all Raman spectra used in this study displayed HD intensities > 50 counts, thereby avoiding false HD peak identification and associated errors in apparent height determination.

The structure of the uploadable data files is described in the Supplementary Information. Please find the complete repository including python scripts via https://github.com/olivierbrcknr/charm.

## Results

### Characterization of reference material and fitting of calibration polynomials

**Fig. 1:**
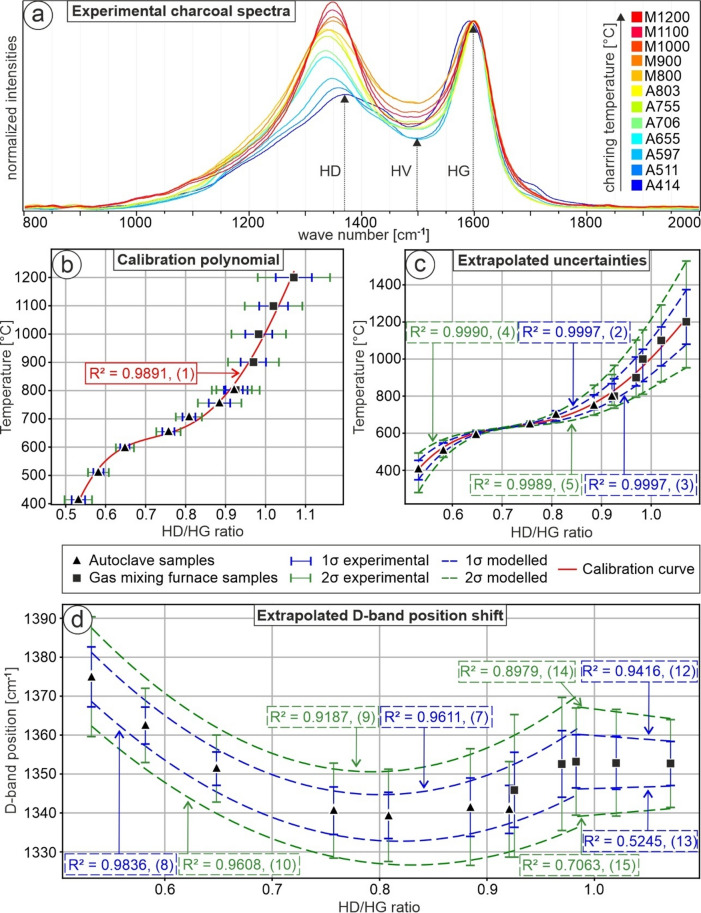
Calibration data based on synthetic amorphous carbon samples derived from pine wood [Eqs. (1–15) refer to polynomial functions in this manuscript]. (**a**) Normalized Raman spectra (colored) of samples experimentally generated between 414 and 1200 °C, displaying the heights of the D-band (HD), V-valley (HV), and G-band (HG). (**b**) The calibration polynomial function (red) describes the evolution of HD/HG ratios over synthetization temperatures. (**c**) Extrapolated temperature uncertainties. Note that the blue 1σ and the green 2σ dashed lines were calculated using the standard deviations from (b) and the calibration polynomial function (**a**). (**d**) Shift of D-band position over synthetization temperature with extrapolated blue 1σ and green 2σ. Note that the D-band position has stabilized for samples produced at ≈ 1000 °C and above.

As a first step, we generated charcoal samples from pine wood both in an autoclave setup (T = 414, 511, 597, 655, 706, 755, and 803 °C) and in a 1-atm gas mixing oven setup (T = 800, 900, 1000, 1100, and 1200 °C). For each specific temperature-generated sample (Fig. 1), 123 to 198 Raman spectra were taken and subsequently de-noised and baseline-corrected as detailed in the methodological description to obtain the D-band, V-valley, and G-band values and to calculate the HD/HG ratios and all respective standard deviations (SDs) (Table 1). Generally, the overall intensity of the Raman spectra decreases with increasing charring temperatures of the samples.

**Table 1:** List of samples from the autoclave (A) and 1-atm gas mixing oven (M) setup synthesized at given temperatures (T). Raman spectroscopy data comprises the number of measurements (n), the HD/HG ratios, and the D-band positions with the respective standard deviations (SD).

Name	T [°C]	*n*	D-band [cm^− 1^]	SD [cm^− 1^]	V-valley [cm^− 1^]	SD [cm^− 1^]	G-band [cm^− 1^]	SD [cm^− 1^]	HD/HG	SD
A414	414 ± 5	181	1375	8	1438	57	1595	3	0.5318	0.0172
A511	511 ± 5	181	1362	5	1497	8	1602	1	0.5819	0.0130
A597	597 ± 5	185	1351	4	1503	8	1604	1	0.6482	0.0114
A655	655 ± 5	123	1341	6	1496	12	1603	1	0.7572	0.0154
A706	706 ± 5	180	1339	6	1491	14	1605	1	0.8084	0.0163
A755	755 ± 5	128	1341	8	1488	16	1604	2	0.8850	0.0272
A803	803 ± 5	179	1341	6	1486	15	1604	2	0.9211	0.0221
M800	800 ± 5	176	1346	10	1492	17	1600	4	0.9256	0.0299
M900	900 ± 5	185	1353	9	1490	16	1601	3	0.9696	0.0319
M1000	1000 ± 5	179	1353	7	1488	15	1603	3	0.9827	0.0340
M1100	1100 ± 5	188	1353	7	1487	16	1602	3	1.0201	0.0360
M1200	1200 ± 5	198	1353	6	1484	14	1602	3	1.0708	0.0452

The charcoal generation temperature can be described as a function of the respective HD/HG ratio (x), which results in the following calibration polynomial (Fig. 1b):

1 $$T{\text{ }}[^\circ C]{\text{ }}={\text{ }} - 23618.330{x^4}\,+\,87116.876{x^3} - \,114789.756{x^2}\,+\,65442.100x{\text{ }} - 13145.532;{\text{ }}{R^2}\,=\,0.9891$$

To describe the temperature uncertainty of the calibration polynomial, the SDs of the HD/HG ratios are used to calculate the respective uncertainties (σ, -σ, 2σ, −2σ) with Eq. (2, Fig. 1c). These values are then used to extrapolate the temperature uncertainties as confidence interval polynomials (Eqs. 2–5, Fig. 1c):

2 $${T_\sigma }[^\circ C]{\text{ }}={\text{ }} - 21709.095{x^4}\,+\,80525.133{x^3} - \,105258.023{x^2}\,+\,59163.820x\, - \,11616.731;{\text{ }}{R^2}\,=\,0.9997$$

3 $${T_{ - \sigma }}[^\circ C]{\text{ }}={\text{ }} - 27970.485{x^4}\,+\,101514.384{x^3} - \,133522.558{x^2}\,+\,76495.229x\, - \,15599.193;{\text{ }}{R^2}\,=\,0.9997$$

4 $${T_2}_{\sigma }[^\circ C]{\text{ }}={\text{ }} - 23031.181{x^4}\,+\,83958.914{x^3} - \,107224.811{x^2}\,+\,58693.489x\, - \,11182.297;{\text{ }}{R^2}\,=\,0.9990$$

5 $${T_{ - 2\sigma }}[^\circ C]{\text{ }}={\text{ }} - 34183.205{x^4}\,+\,122106.176{x^3} - \,159827.172{x^2}\,+\,91613.017x\, - \,18866.042;{\text{ }}{R^2}\,=\,0.9989$$

We calculate the temperature-dependent uncertainty as half the difference between the upper and lower 2σ polynomials (Eqs. 4, 5; Fig. 1c) for given HD/HG ratios, intentionally overestimating the uncertainty. Moreover, temperature values are rounded to one decimal place, with associated errors always rounded up. This approach accounts for measurement errors in the temperature during the generation of the calibration charcoal, uncertainties in the Raman spectroscopy measurements, and model fitting uncertainty.

With increasing temperature, the D-band position decreases to 1339 cm^− 1^ up to 706 °C and then increases to 1353 cm^− 1^ up to 900 °C, where it remains constant up to 1200 °C (Fig. 1a, d; Table 1). The characterization of the D-band values and their shifts is used to compare our charcoal calibration to charcoal produced by volcanic and anthropogenic processes. The D-band shift can be described as two sets of polynomials. Based on HD/HG ratios, set 1 is defined with HD/HG ratios ranging from 0.5318 to 0.9840, whereas set 2 has HD/HG ratios ranging from 0.9840 to 1.0708. This threshold was chosen to optimize the R^2^ values of the polynomials. For set 1, the polynomial describing the mean D-band position as a function of the respective HD/HG ratio (x) is:

6 $$D{\text{ }}\left[ {c{m^{ - 1}}} \right]{\text{ }}={\text{ }}470.652{x^2} - {\text{ }}761.512x{\text{ }}+{\text{ }}1646.797;{\text{ }}{R^2}={\text{ }}0.9841$$

The corresponding extrapolated SDs of the D-band shift are (Eqs. 7–10, Fig. 1d):

7 $${D_\sigma }[c{m^{ - \,1}}]\,=\,504.776{x^2} - \,808.431x\,+\,1668.396;{\text{ }}{R^2}\,=\,0.9611$$

8 $${D_{ - \sigma }}[c{m^{ - \,1}}]\,=\,436.529{x^2} - \,714.593x\,+\,1625.197;{\text{ }}{R^2}\,=\,0.9836$$

9 $${D_2}_{\sigma }[c{m^{ - \,1}}]\,=\,538.900{x^2} - \,855.350x\,+\,1689.995;{\text{ }}{R^2}\,=\,0.9187$$

10 $${D_{ - 2\sigma }}[c{m^{ - \,1}}]\,=\,402.405{x^2} - \,667.674x\,+\,1603.598;{\text{ }}{R^2}\,=\,0.9608$$

For set 2, the polynomial for the D-band position mean as a function of the respective HD/HG ratio (x) is:

11 $${\text{D }}\left[ {{\mathrm{cm}}^{{ - {\mathrm{1}}}} } \right]{\text{ }} = {\text{ }} - {\mathrm{5}}.{\text{747x }} + {\text{ 1358}}.{\mathrm{839}},{\text{ R}}^{2} {\text{ }} = {\text{ }}0.{\mathrm{9275}}$$

The corresponding extrapolated SDs of the D-band shift are (Eqs. 12–15, Fig. 1d):

12 $${D_\sigma }[c{m^{ - \,1}}]{\text{ }}={\text{ }} - 20.142x\,+\,1380.0349;{\text{ }}{R^2}\,=\,0.9416$$

13 $${\mathrm{D}}_{{ - \sigma }} \left[ {{\mathrm{cm}}^{{ - {\mathrm{1}}}} } \right]{\text{ }} = {\text{ 8}}.{\text{647x }} + {\text{ 1337}}.{\mathrm{642}};{\text{ R}}^{2} = {\text{ }}0.{\mathrm{5245}}$$

14 $${\mathrm{D}}_{{{\mathrm{2}}\sigma }} \left[ {{\mathrm{cm}}^{{ - {\mathrm{1}}}} } \right]{\text{ }} = {\text{ }} - {\mathrm{34}}.{\text{536x }} + {\text{ 14}}0{\mathrm{1}}.{\mathrm{231}};{\text{ R}}^{2} {\text{ }} = {\text{ }}0.{\mathrm{8979}}$$

15 $${\mathrm{D}}_{{ - {\mathrm{2}}\sigma }} \left[ {{\mathrm{cm}}^{{ - {\mathrm{1}}}} } \right]{\text{ }} = {\text{ 23}}.0{\text{41x }} + {\text{ 1316}}.{\mathrm{446}},{\text{ R}}^{2} {\text{ }} = {\text{ }}0.{\mathrm{7}}0{\mathrm{63}}$$

### Characterization of different wood species

**Fig. 2:**
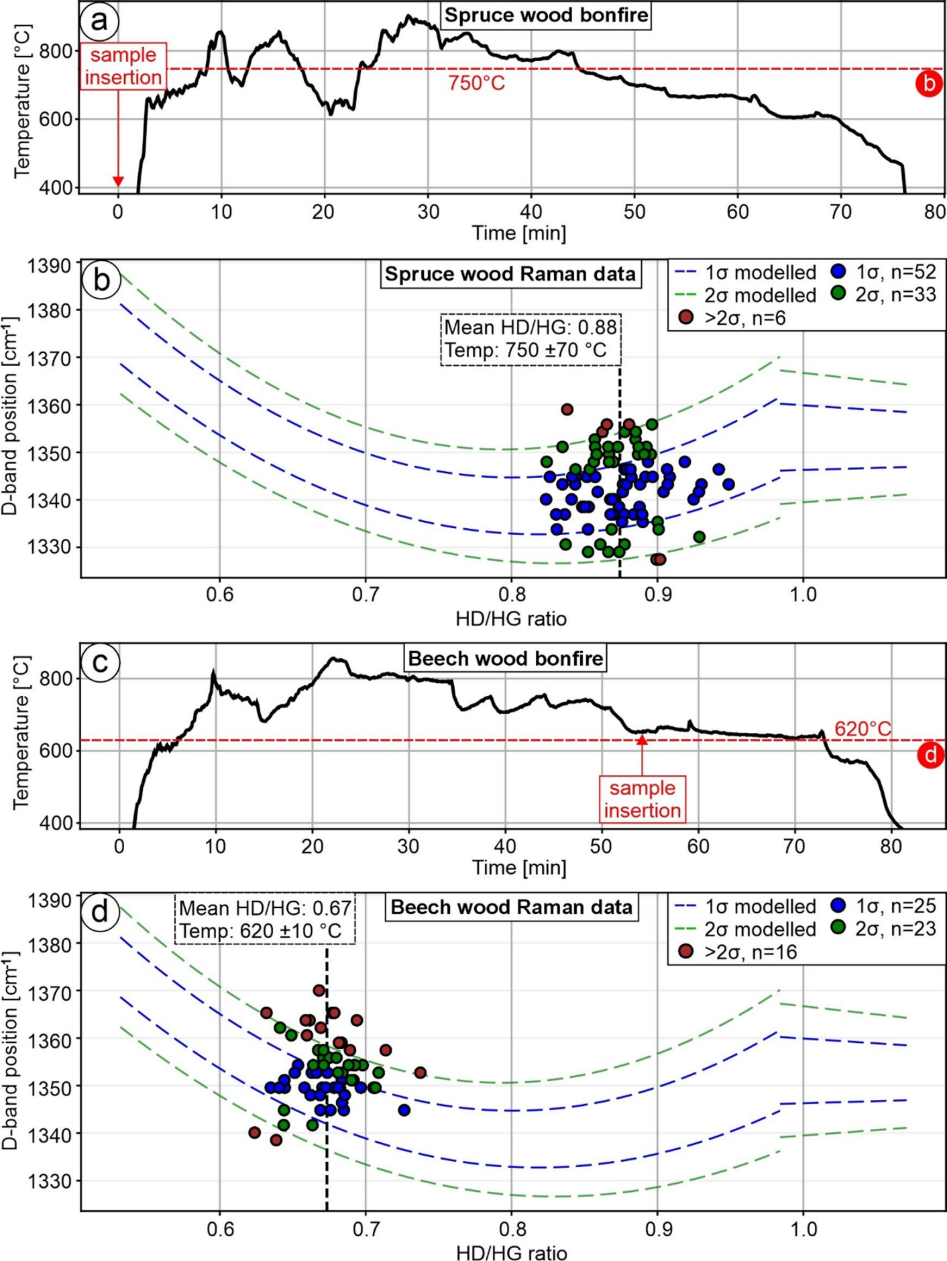
Synthetization of amorphous carbon samples from spruce and beech wood in bonfire-conditions and subsequent analysis. Note that the data point coloring and the dashed lines refer to the comparison to the standard deviation of our calibration. (**a**) Temperature profile recorded throughout the firing process of spruce wood. The red dashed line represents the temperature calculated from the corresponding Raman measurements shown in (**b**), using Eqs. (1, 4, 5). (**b**) Distribution of D-band positions obtained from Raman spectra of spruce wood samples compares well with the calibration data and temperature conditions from (**a**). (**c**) Temperature profile recorded during the firing of beech wood. The red dashed line marks the temperature values inferred from the Raman analysis presented in (**d**), based on Eqs. (1, 4, 5). (**d**) Variation in D-band positions from Raman measurements of beech wood compares well with the calibration data and temperature conditions from (**c**).

To test our calibration with different wood species, we produced bonfires using softwood (spruce; Fig. 2a, b) and hardwood (beech; Fig. 2c, d). The temperature at the bonfire center was monitored with a thermocouple, which was inserted before ignition. Additional logs were added to maintain the fires for more than one hour.

Both softwood and hardwood bonfires reached peak temperatures of approximately 850 °C within the first 10 min. During the firing process, temperatures could intermittently increase, reaching up to 900 °C for short durations (Fig. 2a, c). For the softwood setup, we analyzed charcoal produced from spruce wood used to ignite the fire, which therefore experienced the full heating history (Fig. 2a). In total, 91 data points have a mean HD/HG ratio of 0.8742 (SD = 0.0269), and their mean D-band is 1343 cm^− 1^ (SD = 7 cm^− 1^). When applying calibration Eq. (1) and uncertainty Eqs. (4, 5), a temperature of 750 ± 70 °C is interpreted (Fig. 2b). The result compares well with the maximum temperatures in the experimental setup (Fig. 2a), as well as with the SD of the HD/HG ratio, the mean D-band position, and its SD for the pine wood reference material (Sample A755; Fig. 2b; Table 1). For the hardwood setup, we investigated charcoal produced from beech wood, which was placed next to the thermocouple ≈ 55 min after ignition, at a temperature of about ≈ 640 °C. Raman spectroscopy data of 64 spectra reveal a mean HD/HG of 0.6734 (SD = 0.0222), and their mean D-band is 1353 cm^− 1^ (SD = 7 cm^− 1^). When applying calibration Eq. (1) and uncertainty Eqs. (4, 5), the estimated charring temperature is 620 ± 10 °C (Fig. 2d). The result agrees well with the maximum temperatures in the experimental setup (Fig. 2c). However, the SD of the HD/HG ratio as well as the mean D-band position, and its SD for the pine wood reference material are slightly blue-shifted (upshifted) (Samples A597 and A655; Fig. 2c; Table 1).

### Characterization of charcoal formed in a silicate environment

**Fig. 3:**
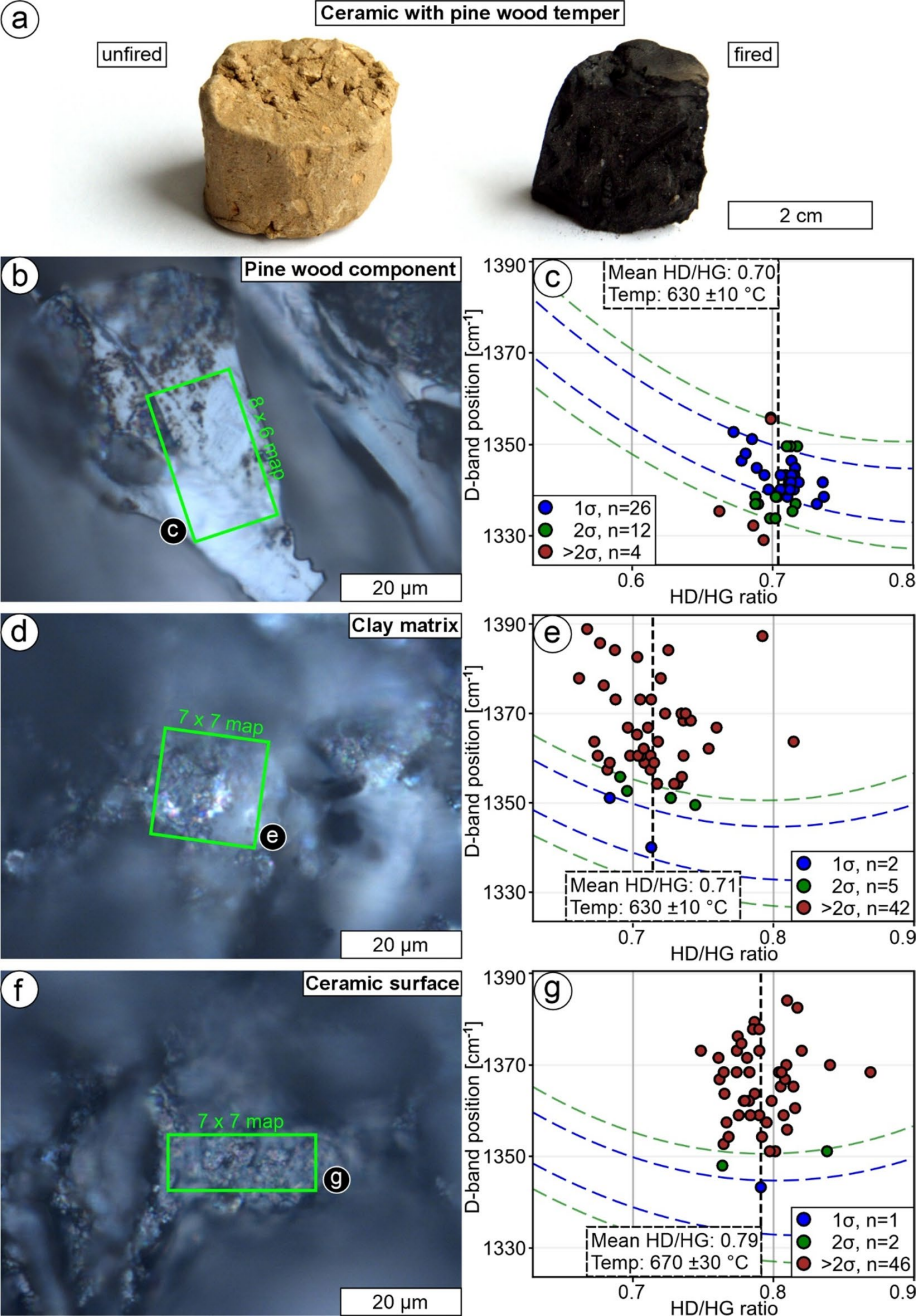
Characterization of a ceramic sample with pine wood temper. Note that the data point coloring and the dashed lines in (**c**, **e**, **g**) refer to the comparison to the standard deviation of our calibration. (**a**) Comparison of unfired clay with fired ceramic. (**b**) Raman spectroscopy map (8 × 6 datapoints) of pine wood component with results in (**c**); reflected light micrograph. (**c**) Variations of D-band positions are consistent with the calibration data. (**d**) Raman spectroscopy map (7 × 7 datapoints) of amorphous carbon in the clay matrix with results in (**e**); reflected light micrograph. (**e**) Variations of D-band positions are commonly higher (> 2σ) compared to the calibration data; however, the estimated temperature based on our calibration is consistent with (**b**, **c**). (**f**) Raman spectroscopy map (7 × 7 datapoints) of amorphous carbon at the ceramic surface with results in (**g**); reflected light micrograph. (**g**) Variations of D-band positions are commonly higher (> 2σ) compared to the calibration data.

To investigate charcoal formation in a silicate environment, we produced a ceramic sample using pine wood as a temper, consistent with our calibration procedure. After firing, the initially brown sample turned black both on the surface and throughout its interior (Fig. 3a). Raman data of 42 spectra of charred pine wood residues within the ceramic reveal a mean HD/HG ratio of 0.7042 (SD = 0.0158) and a mean D-band position of 1341 cm^− 1^ (SD = 6 cm^− 1^; Fig. 3b, c). When applying calibration Eq. (1) and uncertainty Eqs. (4, 5), a temperature of 630 ± 10 °C is calculated (Fig. 3c). The results agree well with the SD of the HD/HG ratio, the mean D-band position, and its SD for the pine wood reference material (Samples A597 and A655; Table 1; Fig. 3c). Raman spectra of the clay matrix obtained from a freshly fractured surface (Fig. 3d) have a pronounced signal from amorphous carbon. A data set of 49 spectra has a mean HD/HG ratio of 0.7141 (SD = 0.0296) and a mean D-band position of 1365 cm^− 1^ (SD = 11 cm^− 1^). When applying calibration Eq. (1) and uncertainty Eqs. (4, 5), the inferred charring temperature is 630 ± 10 °C (Fig. 3e). The temperature agrees with the temperature determined for the pine wood temper (Fig. 3b, c); however, the D-band position and its SD are blue-shifted compared to the pine wood reference material (Sample A655; Table 1; Fig. 3e). Raman spectra of the ceramic surface (Fig. 3f) also have a pronounced signal from amorphous carbon. A data set of 49 spectra has a mean HD/HG ratio of 0.7910 (SD = 0.0229) and a mean D-band position of 1365 cm^− 1^ (SD = 9 cm^− 1^). When applying calibration Eq. (1) and uncertainty Eqs. (4, 5), a temperature of 670 ± 30 °C is inferred (Fig. 3g). This temperature is higher than temperatures calculated from the pine wood residue and the signal from the clay matrix, both from within the ceramic. Moreover, the D-band position and its SD are blue-shifted compared to the pine wood reference material (Samples A655 and A706; Table 1; Fig. 3g).

### Characterization of charcoal in thin section

**Fig. 4:**
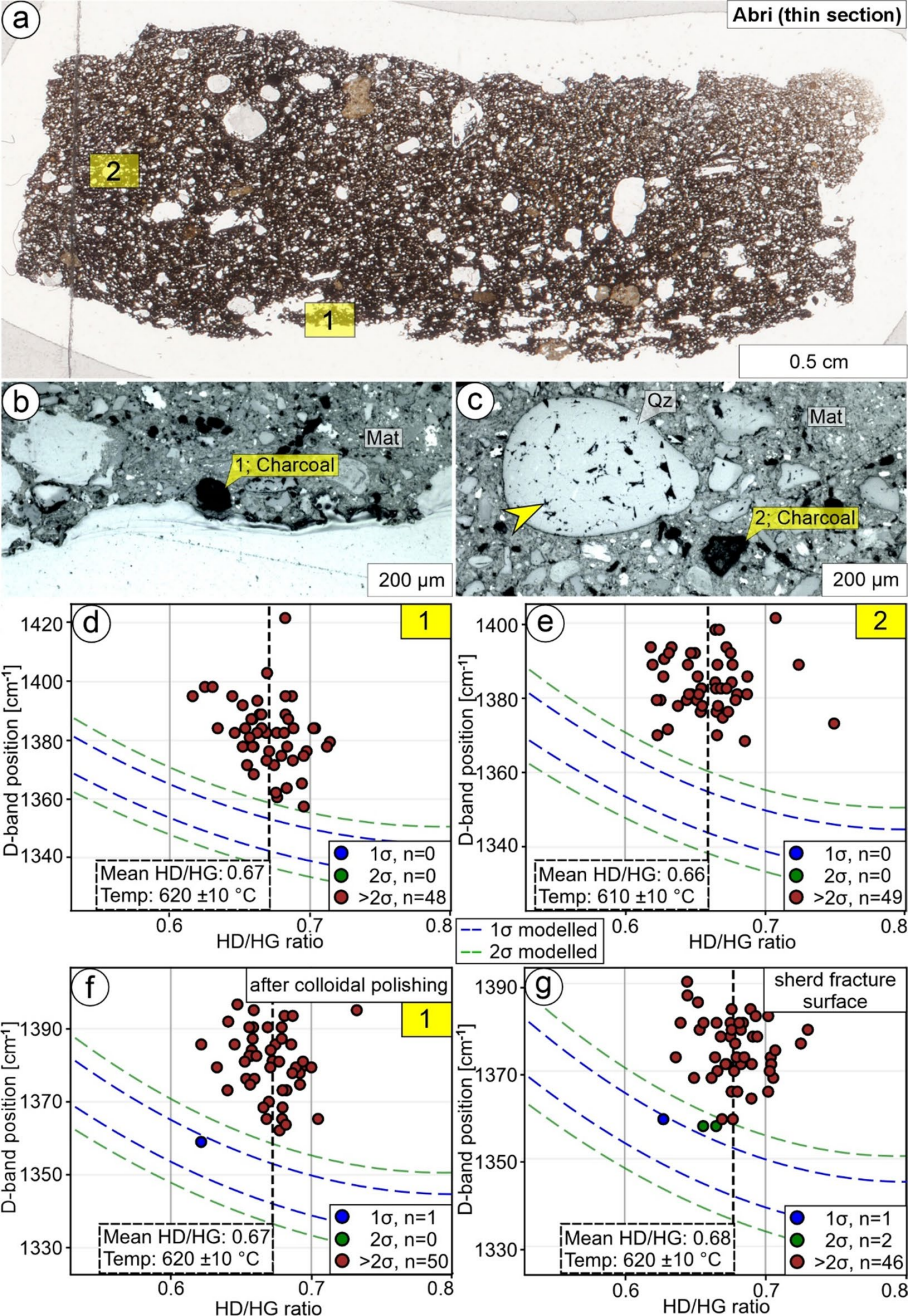
Characterization of charcoal in the recently produced ceramic sample Abri. Note that the data point coloring and the dashed lines refer to the comparison to the standard deviation of our calibration. (**a**) Overview of a thin section with two investigated areas 1 and 2, magnified in (**b**) and (**c**), respectively; micrograph taken with transmitted light. (**b**,** c**) Charcoal fragments (Char) inside the clay matrix (Mat) with adjacent quartz grains (Qz), which have fractures due to thin section preparation (yellow arrow); reflected light micrographs. (**d**,** e**) Raman spectroscopy data of charcoal fragments displayed in (**b**) and (**c**), respectively. Variations of D-band positions are commonly higher (> 2σ) compared to the calibration data. (**f**) Raman spectroscopy data of the same charcoal fragment as in (**b**) and (**d**) after colloidal polishing of the thin section surface. Note the strong similarity in the data sets presented, indicating that surficial polishing during thin section preparation does not obscure the charcoal data. (**g**) Raman spectroscopy data obtained directly from a sherd fracture surface of sample Abri, which is consistent with the data from (d-f), demonstrating that thin section production does not significantly affect the chosen Raman parameters.

To assess the influence of sample preparation and polishing, we investigated and compared a thin section and a sherd from ceramic sample Abri (Fig. 4a). Within its matrix, charcoal fragments with ≈ 50 μm in diameter occur together with quartz grains with diameters up to 400 μm (Fig. 4b, c). In our case, fractured quartz grains (Fig. 4c) resulted from excessive force applied during thin section preparation^45^, which provides an ideal test case for understanding potential sample preparation influences on charcoal Raman spectra. We analyzed charcoal fragments from both the core and the rim (Fig. 4a-c).

The data set of the charcoal from within the rim (Fig. 4b) consists of 48 spectra with a mean HD/HG ratio of 0.6708 (SD = 0.0213) and a mean D-band position of 1382 cm^− 1^ (SD = 12 cm^− 1^). When applying calibration Eq. (1) and uncertainty Eqs. (4, 5), a temperature of 620 ± 10 °C is inferred (Fig. 4d). However, the D-band position and its SD are blue-shifted compared to the pine wood reference material (Samples A597 and A655; Table 1; Fig. 4d). A data set of 49 spectra of the charcoal from within the core (Fig. 4c) has a mean HD/HG ratio of 0.6591 (SD = 0.0261) and a mean D-band position of 1384 cm^− 1^ (SD = 8 cm^− 1^). When applying calibration Eq. (1) and uncertainty Eqs. (4, 5), the interpreted charring temperature is 610 ± 10 °C (Fig. 4e). Again, the D-band position and its SD are blue-shifted compared to the pine wood reference material (Samples A597 and A655; Table 1; Fig. 4e). We then applied a colloidal polishing to the thin section surface, which removes the nanometer-thick amorphous layer, resulting from thin section preparation, which is discussed to obscure structural information^46^ as obtained from Raman spectroscopy. The same charcoal from within the rim (Fig. 4b) was chosen for mapping and resulted in a data set of 51 spectra with a mean HD/HG ratio of 0.6725 (SD = 0.0260) and a mean D-band position of 1381 cm^− 1^ (SD = 10 cm^− 1^). When applying calibration Eq. (1) and uncertainty Eqs. (4, 5), a temperature of 620 ± 10 °C is calculated (Fig. 4f). As with the data set before colloidal polishing, the D-band position and its SD are blue-shifted compared to the pine wood reference material (Samples A597 and A655; Table 1; Fig. 4d). From a sherd from sample Abri, a fracture surface was investigated and resulted in a data set of 49 spectra, which have a mean HD/HG ratio of 0.6771 (SD = 0.0217) and a mean D-band position of 1374 cm^− 1^ (SD = 8 cm^− 1^). When applying calibration Eq. (1) and uncertainty Eqs. (4, 5), the charring temperature is interpreted as 620 ± 10 °C (Fig. 4g). The D-band position and its SD are blue-shifted compared to the pine wood reference material (Samples A597 and A655; Table 1; Fig. 4g).

### Characterization of charcoal produced from a pyroclastic density current

**Fig. 5:**
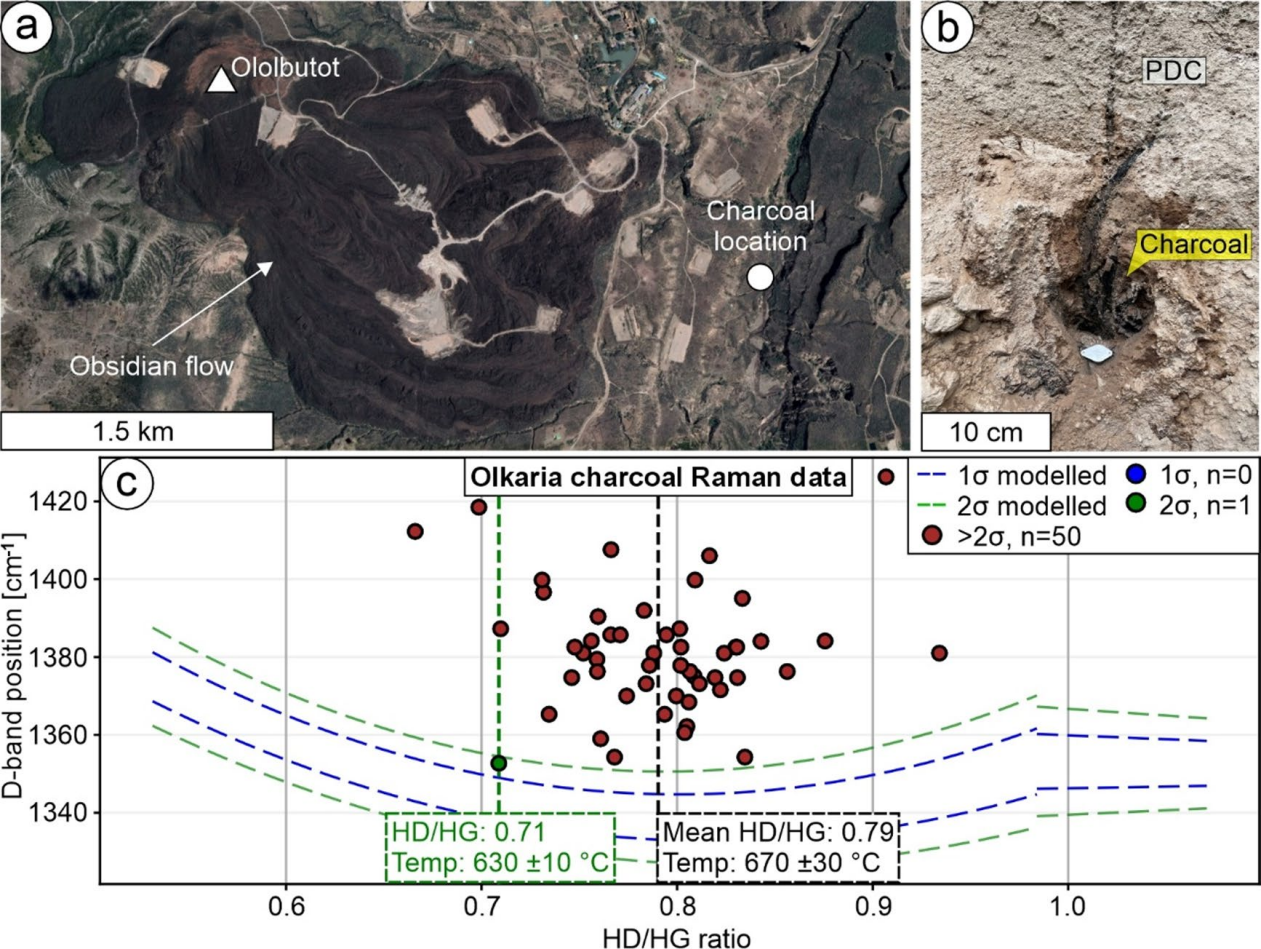
Charcoal generated as a result of an eruption from the Ololbutut vent (Olkaria volcanic complex) at 1759 ± 23 CE in Kenya. (**a**,** b**) Overview of the Ololbutot vent, where an obsidian flow was generated, and charcoal was formed within and underneath a pyroclastic density current (PDC) deposition. (**c**) Raman spectroscopy data of the charcoal displayed in (**b**). Note that the data point coloring and the dashed lines refer to the comparison to the standard deviation of our calibration. Variations of D-band positions are commonly higher (> 2σ), and HD/HG ratios are scattered more compared to the calibration data. Therefore, temperature estimations were conducted with the lowest HD/HG ratio (green), which is comparable with the calibration data. The satellite image in (**a**) was obtained from Google Earth software 7.3 (https://www.google.de/earth/index.html). Imagery 2025 provided by Airbus, CNES/Airbus, Maxar Technologies. Imagery date: 11-07-2024; accessed 11-03-2025; centered at 0°54’17"S 36°17’34"E.

Charcoal from the Olkaria Volcanic Complex was formed as a result of the youngest eruptive phase of a series of recent dome-building and collapse events within the central part of the Olkaria Volcanic Complex^10^(Fig. 5a). The presence of the charcoal at the base of the PDC deposit (Fig. 5b) indicates that an explosive eruption triggered a PDC that incinerated vegetation in the area, preserving the charcoal beneath the pyroclastic material^10^. Charcoal is reported to experience oxidative weathering over time^33^, therefore, the sample from Olkaria provides an ideal test case for studying altered natural charcoal. The data set comprised 51 spectra with a mean HD/HG ratio of 0.7889 (SD = 0.0495) and a mean D-band position of 1381 cm^− 1^ (SD = 16 cm^− 1^). When applying calibration Eq. (1) and uncertainty Eqs. (4, 5), a temperature of 670 ± 30 °C is inferred (Fig. 5c). However, the D-band position and its SD are blue-shifted compared to the pine wood reference material (Samples A655 and A706; Table 1; Fig. 5c). To discuss the effect of oxidative weathering, which is reported to increase the HD/HG ratio^23^, we examined a data point lying within the expected D-band position range of our pine wood reference material, with an HD/HG ratio of 0.7088 and a D-band position of 1354 cm^− 1^. When applying calibration Eq. (1) and uncertainty Eqs. (4, 5), a temperature of 630 ± 10 °C is calculated for this data point (Fig. 5c).

## Discussion

We first discuss our calibration polynomials (Fig. 1) and compare them to the reported structural evolution of charcoal with increasing charring temperature. Next, we discuss our test cases involving different wood types produced in bonfire settings (Fig. 2) and the analysis of charred wood in ceramics relevant in an archaeological context (Fig. 3). We then examine the influence of thin section preparation (Fig. 4) and, finally, evaluate the effect of oxidative weathering on charcoal formed in a PDC deposit (Fig. 5), interpreting the corresponding charring temperature range.

### Comparison of the calibration polynomial with the structural evolution of charcoal during heating

We generated 12 charcoal samples from pine wood in the temperature range 414–1200 °C and subsequently acquired Raman spectroscopy data at ambient temperature. As a result, we fitted the HD/HG ratios of the data sets with an R^2^ of 0.9891 (Eq. 1; Fig. 1b). Within the experimental temperature range, the calibration polynomial has an inflection point at 0.721, corresponding to a temperature of 662 °C. Generally, cellulose-based materials undergo significant thermal decomposition as temperatures rise from 400 to 600 °C. This process breaks down these organic components and leads to a marked increase in aromaticity, fundamentally altering the structure of the carbon present, which is composed of tenths-of-nanometer-sized polyaromatic layers^15,47,48^. The thermal decomposition and increase in aromaticity are likely reflected in our calibration polynomial below an HD/HG ratio of 0.721. In our calibration data, the D-band position decreases to 1339 cm^− 1^ up to 706 °C and then increases to 1353 cm^− 1^ up to 900 °C, where it remains constant up to 1200 °C (Fig. 1a, d; Table 1). In the charcoal formation process, the loss of heteroatoms is almost completed with increasing temperatures up to 1000 °C^28^. At temperatures exceeding 1000 °C, the carbon structure continues to become more ordered and stable, with a reduction in interlayer defects of aromatic stacks^28^, which might explain the temperature-dependent systematic variability of the D-band position up to temperatures of ≈ 1000 °C. Generally, the Raman spectra intensities decrease with increasing charring temperatures of the samples^29^, which likely contributes to the increase in SD (Table 1; Fig. 1c). As a result, the temperature uncertainty increases with charring temperature, consistent with the calibration study for the laser excitation wavelength of 514.5 nm^15^.

For applying our calibration (Eq. 1; Fig. 1b) as a paleothermometer, the influence of residence time at a given highest treatment temperature must be considered. Carbonization, follows kinetic laws; thus, longer residence times can enhance the degree of carbonization by facilitating the release of volatile compounds and potentially altering the calibration curves. To evaluate this effect, the calibration study using a 514.5 nm excitation wavelength^15^ heated pine wood for 1, 6, and 12 h. Regardless of residence time, the HD/HG ratio curves maintained a sigmoidal shape, though data scatter decreased with longer heating durations. The effect is most pronounced between 600 °C and 800 °C, where differences between mean curves are maximal, but becomes negligible above 1000 °C. After 12 h, average HD/HG ratios converge, indicating that in the 600–800 °C range, carbonization mainly involves the gradual removal of volatile matter from nanoporous organic structures^15^.

Consequently, Eq. (1) remains applicable as a paleothermometer for residence times up to 12 h.

### Comparison of calibration with charcoal derived from different wood types in bonfire settings

To evaluate our calibration across different wood species, we conducted individual bonfire experiments using softwood (spruce; Fig. 2a, b) and hardwood (beech; Fig. 2c, d). The investigated charcoal from softwood experienced peak temperatures of ≈ 850 °C, although the observed temperatures strongly varied over the total duration of ≈ 75 min (Fig. 2a). The respective mean HD/HG ratio is 0.8742 (SD = 0.0269) with a mean D-band position consistent with the reference material (Sample A755; Fig. 2b; Table 1). Temperature calculations with Eqs. (1), (4), and (5) result in 750 ± 70 °C (Fig. 2b). Despite slightly higher peak temperatures, the system likely lacked sufficient time to reach thermal equilibrium, which may account for the observed temperature offset. The characterized charcoal from hardwood experienced a temperature plateau of ≈ 630 °C for about 20 min. The respective mean HD/HG ratio is 0.6734 (SD = 0.0222) with a slightly blue-shifted D-band position compared to the reference material (Samples A597 and A655; Fig. 2d; Table 1). The higher SD compared to the reference material (Table 1) is likely due to the shorter carbonization time, which increases the scatter in the Raman data^15^. Temperature calculations with Eqs. (1, 4, 5) result in 620 ± 10 °C (Fig. 2d) and are consistent with the temperature measurements (Fig. 2c). Our results indicate that the pine wood reference material and our methodological approach (Table 1; Fig. 1) applies to spruce and beech wood (Fig. 2), consistent with the 514.5 nm calibration study, which documented, that differences in HD/HG ratios due to precursor material are negligible^15^.

### Characterization of charcoal in a ceramic sample: surface analysis provides temperature conditions

We tempered a ceramic sample with pine wood to investigate charcoal formation in a silicate environment (Fig. 3a). Charred pine wood residues in the ceramic have a mean HD/HG ratio of 0.7042 (SD = 0.0158; Fig. 3b, c) and are consistent with our reference material (Samples A597 and A655, Table 1; Fig. 1). Interestingly, the clay matrix has Raman spectra corresponding to amorphous carbon, which have a mean HD/HG of 0.7141 (SD = 0.0296; Fig. 3d, e). Although the SD of the clay matrix is nearly twice that of the pine wood residues, the respective mean HD/HG ratios are very similar. Before firing, the ceramic was brown, but turned black both internally and on its surface after firing (Fig. 3a). This is likely due to the formation of tars and soot^17^, which subsequently dispersed and precipitated throughout the clay matrix, forming deposits characterized as amorphous carbon^49,50^. Both the char residues and the soot and tars in the matrix indicate a maximum firing temperature of 630 ± 10 °C (Fig. 3b-e). However, the mean D-band position of the tars and soot spectra is blue-shifted (Fig. 3e) compared to the char residue (Fig. 3c) and the reference material (Samples A597 and A655; Table 1; Fig. 1). The position of the D-band in the Raman spectra of amorphous carbon is highly sensitive to the size of sp² clusters. As the size of sp² clusters decreases, the D‑band position typically shows a slight blue-shift and its contribution to the spectrum becomes less pronounced relative to that of larger, more ordered aromatic domains, because smaller aromatic clusters weaken the breathing‑mode vibrations of sixfold rings that give rise to the D‑band in well‑ordered sp² networks. At a fixed defect density, this reduction in aromatic cluster size therefore lowers the effective D‑band response, even though defects remain the necessary scattering centers for its activation^20^. Therefore, we suggest that the blue-shifted mean D-band reflects dispersed and precipitated tars and soot particles, which likely have smaller sp² clusters compared to the char residues. Measurements of the ceramic surface indicate a mean HD/HG ratio of 0.7910 (SD = 0.0229) and a blue-shifted mean D-band position compared to the reference material (Table 1). Our calibration Eqs. (1, 4, 5) result in a temperature of 670 ± 30 °C. The blue-shifted mean D-band position is again likely the result of smaller sp² clusters (tars and soot) compared to the reference material. The observed temperature difference between char residues and the tars and soot within the ceramic interior and those present on the surface reflects variations in firing conditions, as the surface was exposed to higher temperatures due to its proximity to the fuel source. Importantly, the recently produced ceramic sample has not undergone oxidative weathering; however, it exhibits blue-shifted D-band positions, which have previously been attributed to oxidative weathering^23^. Therefore, our data suggest that blue-shifted D-band positions do not necessarily indicate oxidative weathering. Moreover, the presence of amorphous carbon Raman signals in matrix analyses of Egyptian- and Nubian-style ceramics from the Bronze Age was argued to be related to ash that was mixed into the ceramic paste before firing^2^. However, the present findings suggest that Raman spectroscopy cannot be used to prove this, as pyrolysis and the accompanying tar and soot production may also generate an amorphous carbon signature.

Our results show that in studies aimed at determining maximum firing temperatures during ceramic production, sample preparation for advanced analytical techniques may not be required. The permanent blackening of pottery by combustion products^51^, including tars and soot, has a structure corresponding to temperature conditions that can be identified by Raman spectroscopy, applying our method. Moreover, ceramics with only mineral tempers could also be investigated, since the blackening of the surface is likely caused by combustion products from the fuel.

### Characterization of charcoal in thin section: no influence from thin section preparation

A thin section (Fig. 4a) and a sherd of ceramic sample Abri were measured and compared to evaluate potential modifications of mean HD/HG ratios and mean D-band positions as a result of sample preparation. Generally, the D-band is highly sensitive to the presence of defects within the carbon structure, such as vacancies, grain boundaries, edges, and substitutional atoms that break the translational symmetry of the sp² lattice and thus activate the D-band via double-resonance Raman processes. For a given sp² cluster size, an increase in such defects enhances the probability of these double-resonance events and thereby increases the D-band intensity, whereas a reduction in aromatic cluster size tends to weaken the underlying breathing-mode contribution even in the presence of defects^52^. Thin section preparation can result in excessive force on the sample surface^45^, resulting in, e.g., fractured quartz grains (Fig. 4c). Therefore, charcoal Raman parameters could also be modified due to the formation of defects. The mean HD/HG ratio of charcoal in the thin section rim is 0.6708 (SD = 0.0213; Fig. 4b, d), compared to 0.6591 (SD = 0.0261; Fig. 4c, e) for charcoal in the core of the thin section. The mean D-band positions of both charcoals appear blue-shifted compared to the reference material (Fig. 4d, e). We applied colloidal polishing to remove the nanometer-thick amorphous layer produced during thin section preparation. The same charcoal within the thin section rim (Fig. 4b) was remeasured, resulting in a mean HD/HG ratio of 0.6725 (SD = 0.0260) and a blue-shifted mean D-band position (Fig. 4f). There was no detectable difference in mean HD/HG ratios, mean D-band positions, and calculated temperatures in the charcoal from the thin section rim before and after colloidal polishing (Fig. 4c, e). Raman spectra measured from an untreated sherd fracture surface of sample Abri have mean HD/HG ratios of 0.6771 (SD = 0.0217) (Fig. 4g), resulting in a temperature calculation of 620 ± 10 °C (Eqs. 1, 4, 5). The mean D-band position is blue-shifted (Fig. 4g) compared to the reference material (Table 1). Comparison of untreated fracture surfaces, thin sections, and colloidal polished surfaces revealed no difference in mean HD/HG ratios and mean D-band positions. Generally, the D-band positions throughout the sample (Fig. 4) are blue-shifted compared to the reference material (Table 1) and are thus likely the result of smaller sp^2^ cluster sizes^20^.

Polishing effects on Raman spectra of carbonaceous materials are well documented for well-ordered graphitic and highly metamorphosed carbonaceous materials, where polishing modifies the uppermost surface and increases the relative contribution of defect bands, resulting in altered D-band intensities and altered band ratios when spectra are collected directly on polished surfaces^31,53^. In contrast, our charcoal samples represent non-graphitizing, strongly disordered amorphous carbon, in which pre-existing structural disorder and nanometer-scale aromatic clustering dominate the Raman signal. In such materials, any additional polishing-related defects are, within the sensitivity of our 532 nm HD/HG ratio-based approach, negligible relative to the inherent disorder and do not produce systematic changes in overall D- and G- band shape, HD/HG ratio, or D-band position.

Our results indicate that neither thin section preparation nor colloidal polishing effects have an influence on the mean HD/HG ratios and the mean D-band positions used to infer temperatures in our method.

### Characterization of charcoal produced from a pyroclastic density current: effects of oxidative weathering and estimated charring temperature range

The investigated charcoal sample from the Olkaria Volcanic Complex (Kenya) was produced during a volcanic eruption from the centrally-located Ololbutot vent, (Fig. 5a, b) carbon-14 dated to 1759 ± 23 CE^10^. The mean HD/HG ratio is 0.7889, with a SD of 0.0495, approximately three times higher than that of the reference material (Samples A655 and A706; Table 1; Fig. 5c). The mean D-band position is blue-shifted compared to the reference material (Samples A655 and A706; Table 1; Fig. 5c). Oxidative weathering is reported to obscure Raman parameters, increasing the HD/HG ratio as well as the D- and G-band positions^23^. Weathering typically begins at exposed surfaces and along pathways such as fractures, rather than occurring uniformly throughout the material. This is likely reflected in the large SD of the HD/HG ratio in sample Olkaria, where not all data points are equally affected. However, we cannot distinguish between the effects of small sp^2^ clustering and oxidative weathering, which both blue-shift the D-band position^20,23^. Among 51 spectra, only one spectrum yields values comparable to the reference material, with an HD/HG ratio of 0.7088 and a D-band position of 1354 cm⁻¹ (Table 1; Fig. 5c). This data point likely represents either unaltered or minimally altered charcoal, thus not or only slightly affected by oxidative weathering. We therefore estimate the temperature range experienced by the charcoal during its formation, using the minimum HD/HG ratio from the spectra comparable to our reference material and the maximum HD/HG ratio based on the data set median, accounting for respective uncertainties (Fig. 5c). As a result, we estimate a range of 620–700 °C. Although oxidative weathering can obscure the Raman spectra of charcoal, statistical analysis of data variability may still yield meaningful temperature estimates.

To date, the internal temperatures of PDCs are poorly constrained, yet are critical for accurately modelling their hazards, interpreting their deposits, and understanding the conditions for thermal destruction or preservation of organic and geologic materials. Charcoal produced during eruptive events, when analyzed with our new method, can offer valuable insights into PDC temperature regimes.

## Conclusions

We present a calibration method for determining charring temperatures based on the mean HD/HG ratios of amorphous carbon, as measured by Raman spectroscopy using a 532 nm laser excitation wavelength. This calibration extends the methodological framework proposed for the 514.5 nm calibration^15^ to the widely used 532 nm configuration and integrates recent insights from oxidative weathering effects^23^. By statistically analyzing our charred pine wood reference material, we established criteria based on both the mean D-band position (± SD) and mean HD/HG ratio (± SD). Since Raman spectroscopy calibrations are sensitive to variations in data processing, we provide with CHARM a free, automated tool (see Methods) that enables users to upload individual Raman spectra or Raman maps for automatic processing—de-noising, baseline correction, and Raman parameter extraction—to ensure consistency with our calibration protocol. Additionally, the tool automatically infers the charring temperature from the processed 532 nm data and enables downloads of Raman parameter lists and data visualization, streamlining the workflow for users. This ensures consistency with our calibration protocol and methodology, drastically reduces data processing time, and allows researchers without specialized knowledge of Raman spectroscopy and subsequent data analysis to use our method effectively.

Based on our test case observations, we draw the following conclusions:

- Blackened ceramic surfaces caused by combustion products can be used to determine firing conditions without the prerequisite of organic tempering and the need for sample preparation.
- Thin section preparation does not modify charcoal Raman parameters, including mean HD/HG ratios and mean D-band positions.
- Statistical analysis of the mean D-band position and mean HD/HG ratio—compared to our reference material—can be used to identify sp² clustering effects and potential oxidative weathering.
- Charcoal modified by oxidative weathering can still be used to estimate likely charring temperature ranges by analyzing the statistical distribution of data points and comparing them to our reference material.

In conclusion, our method provides a robust, wavelength-specific reference for Raman-based temperature estimation of amorphous carbon. It enables reproducible applications in archaeology, volcanology, and related materials studies, and offers particular value to laboratories using 532 nm Raman excitation. Beyond these fields, the approach also opens perspectives for forensic and environmental investigations involving thermally altered carbonaceous materials.

## Supplementary Information

Below is the link to the electronic supplementary material.

Supplementary Material 1

## Data Availability

The source code for data processing for both python- and web-versions are available here: https://github.com/olivierbrcknr/charm.
