# Supplementary material for: Reconstructing charcoal formation temperatures in archaeology and volcanology using an automated 532 nm Raman spectroscopy approach
Source: Sci Rep. 2026 May 23;16:16018. doi: 10.1038/s41598-026-53711-0 (PMC13198549; doi:10.1038/s41598-026-53711-0)
Supplement: Supplementary file 1 — Supplementary Material 1 [file 41598_2026_53711_MOESM1_ESM.docx]

**Raman data upload**

Our tool was established to process files from the HORIBA LabSpec 6 Spectroscopy Suite Software. The exported *.txt data files are structured as described below.

**Single spectra**

The file can begin with a metadata header, where each line starts with a hash symbol (#) and details the experimental conditions and instrument settings. Following the metadata, the spectral data block presents the Raman spectrum as a sequential list of data pairs. Each line contains two numerical values separated by a tabulator: the first represents the Raman shift, and the second the corresponding intensity. These pairs are ordered according to increasing Raman shift, covering the specified spectral range with high resolution. This explicit pairing of shift and intensity ensures unambiguous interpretation and facilitates further processing, including denoising, baseline correction, and band/valley detection. An example of a Raman spectrum file can be found via <https://github.com/olivierbrcknr/charm/blob/main/test_files/test_spectrum.txt>.

**Map**

The structure of a Raman map suitable for automated single spectra extraction is defined by both its data organization and its compatibility with our computational parsing routines. A Raman map is fundamentally a two-dimensional spatial dataset, where each measurement point on the sample surface is associated with a complete Raman spectrum. The data is typically stored in a tab-delimited text file using the ISO-8859-1 character encoding. The file begins with a header row, which lists the reference x-axis values—these represent the Raman shift positions (commonly in cm⁻¹) and are uniform across all spectra in the map. Each subsequent row corresponds to the intensity values of a single spectrum, measured at each x-axis position. This arrangement creates a matrix-like structure where the columns correspond to Raman shift positions and the rows (after the header) correspond to individual measurement locations. Comment lines, which start with a hash symbol (#), are permitted anywhere in the file and are ignored during processing. This allows the inclusion of metadata or annotations without disrupting the data extraction workflow. A critical aspect of this structure is the handling of missing data. If an intensity value is absent for a particular Raman shift in a given spectrum, the corresponding field is left empty—represented by two consecutive tab characters. During processing, such missing values are interpreted as null and omitted from the output. For scientific rigor, each spectrum must contain the same number of fields as the reference x-axis row. This ensures that every intensity value is properly aligned with its corresponding Raman shift, and that the dataset is internally consistent. When processed, the script reads the file line by line, ignoring comments, and parses each row into lists of numerical values. For each spectrum, it pairs the x-axis values with the corresponding intensity values, omitting any pairs where the x-value or intensity is missing. An example of a Raman map file can be found in the via <https://github.com/olivierbrcknr/charm/blob/main/test_files/test_map.txt>.
